# Special Issue “Metal Nanoparticles: From Fundamental Studies to New Applications”

**DOI:** 10.3390/ijms27010007

**Published:** 2025-12-19

**Authors:** Emilia Konował, Anna Modrzejewska-Sikorska

**Affiliations:** Faculty of Chemical Technology, Institute of Chemistry and Technical Electrochemistry, Poznan University of Technology, Berdychowo 4, 60-965 Poznan, Poland; emilia.konowal@put.poznan.pl

Nanotechnology is a branch of science focused on the fabrication and study of structures in which at least one dimension is less than 100 nm. Such particles, known as nanoparticles, can be classified according to their shape, origin, and chemical composition. Despite their much smaller size, nanostructures retain the physicochemical properties of their larger counterparts, while also exhibiting additional features that make them suitable for a wide range of novel applications across various fields. Research conducted over the past two decades has clearly demonstrated that the electromagnetic, optical, antibacterial, and catalytic properties of metal nanoparticles are highly dependent on their shape, size, and tendency to agglomerate. This has driven growing scientific interest in metal nanoparticles and their nanometric compounds.

The reduction in particle sizes to the nanometre scale—and the resulting changes in their directionality and structure—has led to the classification of nanostructures based on their dimensionality. Three fundamental categories are distinguished: 1D, 2D, and 3D. Materials confined to one dimension (1D), i.e., quasi-two-dimensional systems, include aluminium nanoplates, Langmuir–Blodgett films, nanopatterned materials, and structures with enhanced abrasion resistance. Quasi-one-dimensional systems, restricted to two dimensions (2D), comprise carbon nanotubes and nanofibres, oxide and semiconductor nanorods, as well as magnetic nanowires. Quasi-zero-dimensional (3D) systems include fullerenes, colloidal particles, quantum dots, nanopores, and activated carbon [1,2,3].

Unlike microstructures, nanomaterials display a range of distinct physicochemical properties. These arise primarily from their small particle size, shape, degree of agglomeration, and high surface-to-volume ratio [4]. Metallic nanostructures, in particular, are characterised by a large interfacial area and high chemical reactivity. As their specific surface area increases, so does their biological activity, which results from the higher surface energy [5].

Metal nanostructures also exhibit intriguing optical and magnetic properties. The colour change observed in solutions as the dimensions of nanostructures are adjusted results from characteristic optical phenomena that occur at the nanoscale [6]. From a mechanical perspective, their resistance to cracking and abrasion increases because the number of structural defects diminishes with decreasing size [2].

Properties such as hydrophilicity, biocompatibility, creep resistance, adsorption capacity, and plasticity distinguish nanomaterials from macroscopic structures [7]. In addition, they are characterised by their low mass and reduced melting point. Their properties are determined using analytical techniques such as UV–Vis spectroscopy, X-ray diffraction, Fourier transform infrared spectroscopy (FTIR), as well as scanning and transmission electron microscopy.

Nanostructures are obtained using three main approaches [8]: the top-down method, the bottom-up method, and the green chemistry method.

The top-down method involves reducing the dimensions of a material by mechanically processing it to the nanometric scale. Techniques such as grinding, crushing, and lithography are used for this purpose. Grinding is typically performed in ball mills under anaerobic conditions, which enables the production of nanomaterials on an industrial scale. Lithography, which employs UV or X-ray radiation, allows the formation of nanostructures with defined shapes and is widely applied in optoelectronics and integrated circuit fabrication [9].

The bottom-up method, in contrast, involves constructing structures from individual atoms or molecules. This approach includes processes such as gas-phase deposition, electrolytic deposition, sol–gel techniques, molecular beam epitaxy, and redox reactions [2,9]. Nanomaterials produced using this method are characterised by a high purity and homogeneity.

The green chemistry method, developed by Paul Anastas in 1991 as part of the Green Chemistry programme [10], is also gaining importance. It involves, among other aspects, the elimination of toxic solvents, a reduction in by-products, the use of renewable raw materials, the complete consumption of reagents, and low energy consumption [11]. This approach allows the synthesis of nanostructures using biological compounds such as bacteria, enzymes, fungi, algae, and amino acids. Owing to the reducing and stabilising properties of these substances, nanomaterials can be produced in an ecological, efficient, and economical manner. The process generates minimal waste and does not require toxic reagents, making it environmentally friendly.

In the synthesis of gold nanoparticles, which involves the reduction of Au^3+^ ions to metallic gold, environmentally friendly solvents and ecological reducing and stabilising agents are increasingly being employed in accordance with the principles of green chemistry. This role is most often fulfilled by plant extracts containing natural phenolic compounds, alkaloids, and terpenoids [11,12].

The first biosyntheses of AuNPs were carried out using extracts from the leaves of scented geranium (*Pelargonium graveolens*), lemongrass (*Cymbopogon flexuosus*), and tamarind (*Tamarindus indica*). In subsequent years, extracts from many other plants have been used for gold nanoparticle synthesis, including aloe vera (*Aloe barbadensis*), magnolia (*Magnolia kobus*), green tea (*Camellia sinensis*), raisin tree (*Hovenia dulcis*), edible musk (*Abelmoschus esculentus*), seaweed (*Galaxaura elongata*), almond tree (*Terminalia catappa*), and onion (*Allium cepa*) [13,14,15,16,17,18,19,20].

Other biological substances with reducing and stabilising properties are also used for the biosynthesis of AuNPs, including fungi (*Alternaria* sp., *Rhizopus oryzae*, *Aspergillus oryzae*, *Phanerochaete chrysosporium*, and *Penicillium brevicompactum*), bacteria (*Bacillus licheniformis*), as well as lignosulphonates, peptides, enzymes, and polysaccharides such as starch and chitosan [21,22,23,24,25,26,27].

Syntheses based on green chemistry principles offer numerous advantages, including low process costs, environmental friendliness, the absence of toxic waste, the ease of scalability, energy savings, and high economic efficiency [28].

Owing to their non-toxicity, biocompatibility, and high stability, AuNPs are widely applied in medicine, electronics, optics, catalysis, cosmetics, and the food industry [29,30,31].

In biomedicine, gold nanoparticles are employed as carriers for drugs and genes, including the delivery of antimicrobial agents such as kanamycin. The use of AuNPs as biosensors allows for the detection of DNA, proteins, and pathogens [30].

Owing to their strong surface plasmon absorption, AuNPs emit scattered light and heat, enabling applications in TEM and X-ray imaging, diagnostics, and photochemical therapy [32]. In recent years, their role in cancer therapies has been increasing: optically excited nanoparticles effectively destroy cancer cells, and combining their action with cisplatin has been shown to enhance treatment efficacy by up to 78% [33]. Pegylated gold nanoparticles are also gaining recognition as a promising adjunct for prostate cancer radiotherapy.

Not only can they enhance the sensitivity of cancer cells to radiation, but they can also serve as carriers for microRNAs (miRNAs), which regulate cellular responses to treatment. A study investigated the effects of hypofractionated radiotherapy in combination with pegylated gold nanorods (AuNPr-PEG) on the levels of selected miRNAs in prostate cancer cells. The results showed that the effects depended on both the cell type and the therapy applied, and that combining AuNPr-PEG with radiation led to a reduction in the expression of several key miRNAs. These findings suggest that gold nanoparticles may improve the effectiveness of radiotherapy and, in the future, contribute to the development of more personalised treatments for prostate cancer (Figure 1) [34].

Nanostructured gold is also effective in the treatment of rheumatoid arthritis, as it binds to VEGF and inhibits its activity, resulting in reduced pain and inflammation. Additionally, AuNPs are employed to combat parasites (*Toxoplasma gondii*) using laser light in combination with nanoparticles, enabling effective pathogen destruction. Recent reports also indicate that gold nanoparticles influence chromatin organisation in cancer cells in a radiation dose-dependent manner, modulating both DNA damage and repair mechanisms. Understanding these complex interactions may enhance the effectiveness of radiotherapy by using AuNPs to amplify the impact of radiation on cancer cells [35,36,37].

In electrochemistry, AuNPs are employed in biosensors for disease diagnosis (e.g., Parkinson’s disease), pathogen detection, and the determination of metal ions. For example, a lignosulphonate-stabilised AuNP complex can be used as an electrode modifier for detecting mercury and thallium ions. Gold nanoparticles stabilised with starch and NaOH are also used to detect lead(II) and copper(II) ions, enabling the analysis of contaminants in water samples [23,38,39].

Another important application of AuNPs is in the catalysis of reduction reactions. Gold nanoparticles and Au–Ag alloys act as effective catalysts in the reduction of 4-nitrophenol and picric acid in the presence of sodium borohydride, leading to the formation of 4-aminophenol. Among other supports, nanoparticles stabilised with mung bean starch (MBS-AuNPs) are employed for this purpose [40].

Gold nanoparticles synthesised with the participation of sodium starch octenyl succinate (OSA-AuNPs) have demonstrated not only exceptional catalytic properties but also signal enhancement in Raman spectroscopy. By using OSA as both a reducing agent and stabiliser, stable and homogeneous nanoparticles with high surface activity were obtained. OSA-AuNPs efficiently catalysed the reduction in organic dyes such as methylene blue and rhodamine B, as well as food dyes including tartrazine (E102) and azorubine (E122), using sodium borohydride as the reducing agent. In addition, they served as effective signal enhancers in surface-enhanced Raman spectroscopy (SERS), significantly improving detection sensitivity. These results confirm that OSA-AuNPs can serve as versatile tools in both chemical and sensory applications (Figure 2) [41].

The authors of [42] also focused on catalytic properties, demonstrating that the catalytic activity of gold nanostructures strongly depends on their size and surface structure. The smallest nanoparticles (below 3 nm) and atoms located at the corners exhibit the highest catalytic activity. This indicates that precise control over the structure of gold nanostructures can significantly enhance the efficiency of catalytic processes.

Similarly, the catalytic properties of rhenium nanostructures were investigated in [43]. The study showed that caffeine extracted from both coffee beans and used coffee grounds can serve as a natural reducing agent for the environmentally friendly synthesis of rhenium nanoparticles (ReNPs) without the use of toxic reagents. The resulting ReNPs, stabilised by caffeine, displayed high catalytic activity, efficiently converting 4-nitrophenol to 4-aminophenol within 40–60 min.

Selenium nanoparticles (SeNPs) have also demonstrated effective catalytic activity, efficiently reducing methylene blue in the presence of sodium borohydride [44].

The use of metal and metal oxide nanoparticles is increasingly regarded as a promising strategy to address the growing problem of bacterial resistance to antibiotics. Among the various nanoparticles with antibacterial properties, copper oxide nanoparticles (CuO-NPs) have attracted particular attention. Although the antibacterial activity of copper has long been known, recent years have seen intensified research into the effects of copper oxide nanostructures on biological systems (Figure 3) [45].

Silver nanostructures (AgNPs) synthesised using green chemistry methods have attracted significant interest in nanotechnology. These processes employ environmentally friendly reducing and stabilising agents, with silver nitrate (AgNO_3_) being the most commonly used metal precursor, while perchlorate, tetrafluoroborate, or silver sulphate are used less frequently. Plant extracts are the predominant reducing agents. Extracts from eucalyptus species (*Eucalyptus urophylla*, *E. citriodora*, and *E. robusta*) are commonly employed to produce spherical nanoparticles ranging in size from 4 to 60 nm [46]. 

Yasir et al. [47] employed an extract from *Syngonium podophyllum* to synthesise nanostructures with antifungal properties. Gurunathan et al. [48] used an extract from Korean wormwood (*Artemisia princeps*) to obtain spherical AgNPs with diameters of 10–40 nm. In subsequent studies by the same team [49], an extract from narrow-leaved cattail (*Typha angustifolia*) enabled the production of nanoparticles approximately 8 nm in diameter. The reduction of silver ions in these processes was facilitated by phenolic compounds, alkaloids, and steroids.

Lemon extract (*Citrus limon*) yielded stable nanoparticles of various shapes [50]. Maria Arshad et al. [51] used tree of heaven (*Ailanthus altissima*), producing AgNPs with an average size of 80 nm; their formation was associated with the presence of aldehydes and amines.

Numerous other plants have also been employed for AgNP biosynthesis, including *Aloe vera*, *Geranium*, *Boerhaavia diffusa*, *blackberry fruit*, *Phytolacca decandra*, *Hydrastis canadensis*, *Thuja occidentalis*, *Hibiscus*, *Murraya koenigii*, *Ficus benjamina*, *Ficus religiosa*, and *Cistrus creticus* L. [52,53,54,55,56].

In addition to plants, microorganisms can act as reducing and stabilising agents. Among bacteria, the following have been used: *Klebsiella pneumoniae*, *Pseudomonas stutzeri AG259*, *Lactobacillus strains*, *Bacillus licheniformis,* and *Brevibacterium casei* [57,58,59,60].

Among fungi, the following have been employed: *Fusarium oxysporum*, *Ganoderma neo-japonicum Imazeki*, *Aspergillus terreus*, and *Penicillium fellutanum* [61,62].

Biosynthesis using bacteria is inexpensive but slow and difficult to control with respect to particle shape. In contrast, fungi, due to their ability to bioaccumulate metals and the presence of enzymes that reduce silver ions, enable faster and more efficient biosynthesis.

Other reducing agents and stabilisers are also employed in the green synthesis of nanosilver, including enzymes, lignosulphonates, glucose, as well as starch and its derivatives (e.g., dextrin). Using glucose as a reducing agent and starch as a stabiliser allows for the production of AgNPs with diameters of approximately 5 nm [63].

Plant tannins, found in bark, leaves, and fruit, can also act as reducing agents. AgNPs have been synthesised using extracts from chestnut fruit, mangrove leaves, and quebracho, yielding spherical or triangular nanoparticles with sizes ranging from 35 to 70 nm [64].

Silver nanoparticles (AgNPs) have wide-ranging and versatile applications, similar to gold nanoparticles (AuNPs), rhenium nanoparticles (ReNPs), and selenium nanoparticles (SeNPs). Alongside AuNPs, they can be employed as chemical probes for the detection of heavy metal ions. In [65], nanostructured silver and gold nanoparticles synthesised using sodium lignosulphonate were applied for the spectrophotometric determination of selected heavy metal ions (Cd^2+^, Cu^2+^, Co^2+^, Pb^2+^, and Ni^2+^) in model aqueous solutions. This application is possible because AgNPs and AuNPs act as nanochromophores, exhibiting an absorption band at a wavelength (λ_max_) of 400–450 nm for AgNPs and 530 nm for AuNPs. The addition of heavy metal ions induces a colour change in the samples, accompanied by a shift in the UV spectrum. Moreover, an additional broad band appears, indicating the formation of Ag(Au)NP aggregates with the heavy metal ions.

Silver nanostructures can also serve as excellent modifiers of glassy carbon electrodes (GCEs) [66]. The modified electrode (GCE/AgNPs) was used for the determination of trace amounts of thallium ions via stripping anodic voltammetry. Among heavy metals, thallium is one of the most toxic, surpassing mercury, cadmium, lead, copper, and zinc in terms of harmfulness. Its high toxicity is attributed to the chemical similarity of the Tl^+^ ion to the K^+^ ion, as both have comparable radii and charges, allowing thallium to interfere with key biological processes. This study particularly focused on evaluating the impact of surface modification on the electrode’s electrochemical performance. The AgNPs-modified electrode demonstrated several significant advantages, including a wide detection range, reduced analysis time due to the elimination of time-consuming preconcentration steps, and safer operation for both the environment and users compared with traditional mercury electrodes.

The impact of nanostructures on humans and the environment. Scientists worldwide are currently examining the future of nanoscience and its potential impact on humans and the environment. Although nanotechnology offers numerous benefits, the risks associated with its development cannot be ignored. Both users of products containing nanostructures and individuals involved in their production are exposed to their effects. The negative impact may also extend to living organisms and entire ecosystems.

Green chemistry methods help mitigate, but do not entirely eliminate, environmental risks, as nanostructures are similar in size to biomolecules found in organisms. Waste containing nanomaterials can contaminate water and pose a threat to wildlife, particularly fish.

Nanoparticles enter the environment from two types of sources:

- Point sources, such as sewage treatment plants, manufacturing facilities, and waste disposal sites;
- Area sources, resulting from the use of products containing nanostructures [67].

Regardless of their origin, most nanoparticles eventually accumulate in soil or water, either directly or indirectly—for example, through sewage treatment and waste processing [68].

Metal nanostructures can be toxic to living organisms, including mammals and fish. They can enter the human body through the digestive tract, respiratory tract, skin, or parenterally. For example, creams containing nanoparticles smaller than skin pores can penetrate the body. The excessive accumulation of nanometals, such as silver, may lead to health disorders, for instance, argyria, which is manifested by permanent skin discolouration [69].

Studies indicate that silver nanoparticles can negatively affect the nervous system, causing cognitive impairment, and the reproductive system, as well as inducing short-term and working memory disorders. Particles capable of forming aerosols can be inhaled, potentially leading to diseases such as pneumoconiosis or lung cancer.

In aquatic environments, metallic nanoparticles pose a serious threat to both fauna and flora. In fish, they can penetrate the gills or body walls, while in invertebrates, they may disrupt the immune and digestive systems [70].

Substances formed during the degradation of nanomaterials can damage DNA, proteins, and cell membranes, leading to metabolic disorders and disease [68].

In summary, despite the numerous benefits of nanotechnology, its development must be approached with caution and careful control. It is essential to design synthesis processes and products that minimise harm to both the environment and human health.

## Figures and Tables

**Figure 1:**
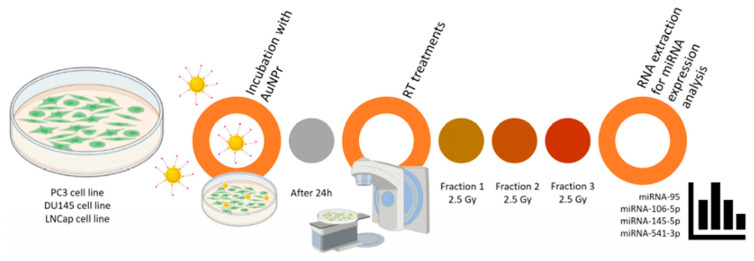
Representative scheme of the workflow for AuNPr-PEG and RT treatment in PC3, DU145, and LNCaP cell lines [34].

**Figure 2:**
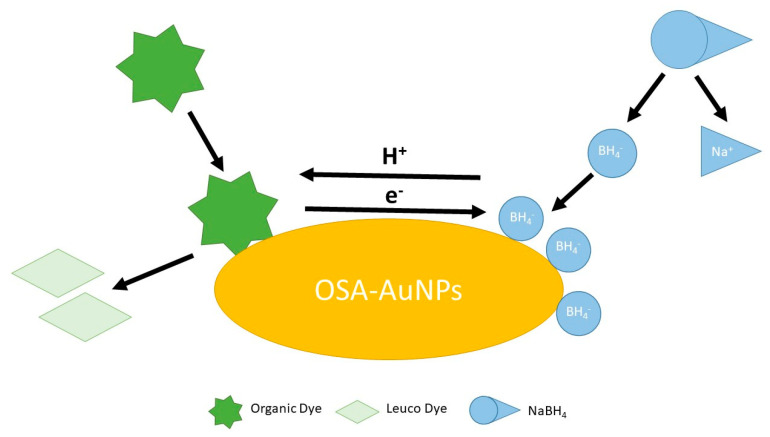
The schematic illustration of the dye decomposition process using OSA−AuNPs(e) [41].

**Figure 3:**
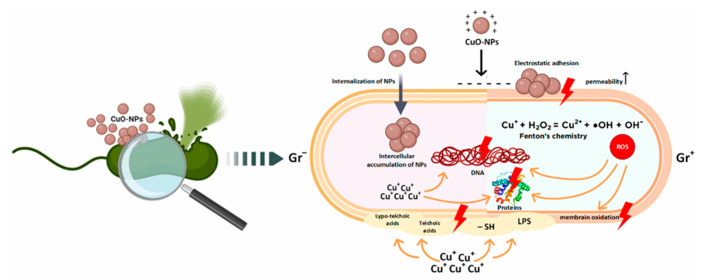
Key mechanisms of antibacterial action of copper oxide nanoparticles according to data from the literature [45].
